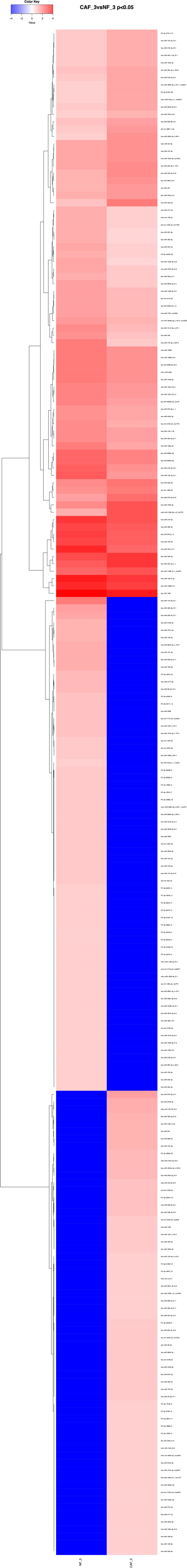# Biological differences between normal and cancer-associated fibroblasts in breast cancer

**DOI:** 10.1016/j.heliyon.2023.e19803

**Published:** 2023-09-06

**Authors:** Dengdi Hu, Wenying Zhuo, Peirong Gong, Feiyang Ji, Xun Zhang, Yongxia Chen, Misha Mao, Siwei Ju, Yuehong Pan, Jun Shen

**Affiliations:** a Affiliated Cixi Hospital, Wenzhou Medical University, Ningbo, 315300, Zhejiang, China; b Affiliated Sir Run Run Shaw Hospital, Zhejiang University School of Medicine, Hangzhou, 310016, Zhejiang, China; c, China (Key Laboratory of Cancer Prevention and Intervention, Ministry of Education), China; d Biomedical Research Center and Key Laboratory of Biotherapy of Zhejiang Province, Hangzhou, 310016, Zhejiang, China

**Keywords:** Breast cancer, Cancer-associated fibroblasts, Normal fibroblasts, Biological profile, microRNA

## Abstract

**Background:**

Cancer-associated fibroblasts (CAFs) constitute the primary constituents of the tumor microenvironment (TME) and exert significant influences on cancer progression. However, adequate comprehension of CAF profiles in breast cancer, as well as the precise mechanisms underlying their promotion of cancer, remains lacking.

**Objectives:**

To discerns the biological differences between normal fibroblasts (NFs) and CAFs in breast cancer and explore the underlying mechanism.

**Methods:**

Three pairs of CAFs and NFs were isolated from breast cancer patients of diverse subtypes who had not undergone prior radiotherapy or chemotherapy. Morphological characteristics of CAFs and NFs were assessed through optical and electron microscopy, their biological attributes were examined using cell counting kits and transwell assays, and their impact on breast cancer cells was simulated using a coculture system. Furthermore, the miRNA profiles of CAFs and NFs were sequenced via an Illumina HiSeq 2500 platform.

**Results:**

CAFs exhibited higher growth rate and motility than NFs and a stronger potential to promote the malignancy of breast cancer cells. RNA sequencing of both NFs and CAFs revealed differentially expressed miRNAs with notable variability among distinct patients within their NFs and CAFs, while the enrichment of the target genes of differentially expressed miRNAs within both GO terms and KEGG pathways demonstrated significant similarity across patients with different profiles.

**Conclusion:**

CAFs have greater malignancy and higher potential to influence the growth, migration, invasion and chemoresistance of cocultured breast cancer cells than NFs. In addition, the miRNAs that are differentially expressed in CAFs when compared to NFs display substantial variability across patients with distinct breast cancer subtypes, while the enrichment of target genes regulated by these miRNAs, within GO terms and KEGG pathways, remains remarkably consistent among patients with varying profiles.

## 1 Introduction

Breast cancer is the most prevalent malignancy among women, contributing to approximately half a million deaths annually [1]. Among the primary causes of refractory breast cancer are treatment resistance and metastasis, and within this context, the tumor microenvironment (TME) has emerged as a central player in fostering both treatment resistance and metastatic processes [2,3]. It is highly complex and heterogeneous, comprising genetically stable noncancerous cells, including fibroblasts, endothelial cells, epithelial cells and immune cells, among which cancer-associated fibroblasts (CAFs) are one of the most abundant components of the TME [4]. It has been postulated that the CAFs in breast cancer originate from diverse sources, from normal fibroblasts (NFs) and mesenchymal stem cells (MSCs) to transdifferentiated adipocytes and pericytes [5]. CAFs can promote tumor drug resistance and mediate tumor metastasis during tumor progression [[6], [7], [8]]. Compared to NFs, CAFs exhibit noteworthy variations in cytokine expression, extracellular matrix (ECM) molecules and cellular metabolism genes, which underpin their pro-tumorigenic properties [[9], [10], [11], [12]].

MicroRNAs (miRNAs) are endogenous RNA molecules of approximately 22 nucleotides in length that regulate target gene expression at the posttranscriptional level. Given the genetic stability of NFs and CAFs [6], we hypothesized that CAFs are posttranscriptionally transformed. In addition, considering that miRNAs are vital for gene expression at the posttranscriptional level, we conducted a comparative analysis of miRNA expression profiles between NFs and CAFs through RNA sequencing to elucidate the distinctions between NFs and CAFs.

## 2 Materials and methods

### 2.1 Tissue samples obtaining and fibroblasts isolation

Human breast tumor tissues were obtained from 3 treatment naïve patients with Luminal A, Luminal B and HER2 positive breast cancer, respectively, after surgery at Cixi Hospital of Wenzhou Medical University (Fig. 1). Their tumor tissues and corresponding grossly normal breast tissues (at least 5 cm away from the tumor) were retrieved and placed in Dulbecco's Modified Eagle's medium (DMEM) with 10% fetal bovine serum (FBS) and 1% penicillin-streptomycin on ice and transported to the laboratory within 30 min after resection. Subsequently, the tissues were washed three times with PBS containing 100 U/ml penicillin and 100 μg/ml streptomycin (Sigma, USA) and minced into fragments of approximately 1 mm^3^. After centrifugation at 2000*g* for 5 min, the supernatant containing adipose tissue was removed, and the precipitated tissues were digested for 4 h at 37 °C in DMEM containing 10% FBS, 2 mg/ml collagenase I (Sigma, St. Louis, MO, USA) and 2 mg/ml hyaluronidase (Solarbio, China). After digestion, the samples were passed through a 100 μm mesh (Biosharp, China) and underwent centrifugation and washing with PBS, following which the cell pellets were resuspended in DMEM with 10% FBS and transferred into 100 mm tissue culture dishes. The CAFs and NFs were routinely maintained in DMEM containing 10% FBS, 100 U/ml penicillin and 100 μg/ml streptomycin. All experimental procedures were conducted using fibroblasts that had undergone fewer than 10 passages to prevent senescence-related effects.

**Fig. 1:**
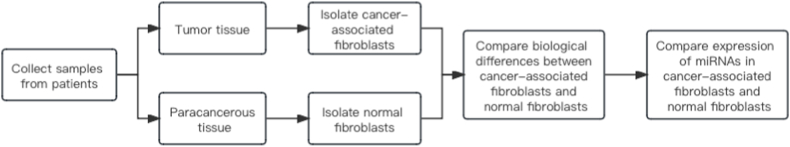
Flowchart of this present study.

### 2.2 Cells and antibodies

Fibroblasts were isolated using a previously described method. Briefly, HCC1937 breast cancer cells (ATCC, USA) were cultivated in RPMI-1640 supplemented with 10% FBS, 100 U/ml penicillin and 100 μg/ml streptomycin and maintained at 37 °C in a humidified environment with 5% CO_2_. The antibodies used for western blotting and immunofluorescence were: αSMA (MAB1420, R&D, USA), Vimentin (5741S, CST, USA) and β-actin (sc-477748, Santa Cruz, USA).

Scanning electron microscopy (SEM) and transmission electron microscopy (TEM) examination

For SEM, mammospheres derived from NFs or CAFs were initially fixed in a solution containing 0.1 M cacodylate buffer (Sigma, USA) supplemented with 2.5% glutaraldehyde (Sigma, USA) and 2% sucrose (Sigma, USA) at a pH of 7.3 at room temperature for 1 h. Mammospheres were washed once with the same cacodylate buffer and then fixed in cacodylate buffer supplemented with 1% osmium tetroxide for 90 min. Mammospheres were washed with cacodylate buffer and then dehydrated with graded concentrations of ethanol. Then, the dehydrated mammospheres were dried using a critical point dryer, coated with gold using the ACE200 coating system (Leica, Germany) and examined using a Nova Nano 450 microscope (Thermo FEI, Czech Republic).

For TEM analysis, the mammospheres from NFs and CAFs were fixed with 2.5% glutaraldehyde for 1 h at room temperature. Subsequently, the samples were post-fixed with 1% osmium tetroxide, dehydrated using graded alcohol concentrations, embedded for sectioning, and subjected to staining with uranyl acetate and lead citrate. Finally, the sections were examined using a Tecnai G2 spirit microscope (Thermo FEI, Czech Republic) for detailed analysis.

### 2.3 Coculture of fibroblasts and tumor cells

Coculture systems were established using Transwell 6-well plates (0.4 μm pores, polyester membrane; Corning, USA). In the upper inserts, NF or CAF cells (1 × 10^5^) were seeded, while 1 × 10^5^ HCC1937 cells were placed in the lower compartment of the culture wells. Following cell attachment, the upper inserts containing fibroblasts were introduced into the wells seeded with HCC1937 cells, and the cell culture medium was replaced with fresh RPMI-1640 without FBS. As a control, HCC1937 cells cultured in RPMI-1640 without FBS were used. After 48-h incubation, the HCC1937 cells were harvested for subsequent experiments.

### 2.4 Immunofluorescence assay

To determine the expression level of αSMA in NFs and CAFs, 3 pairs of NFs/CAFs were seeded on glass slides pre-coated with poly-d-lysine (Sigma-Aldrich, USA). Next, the cells were fixed using 4% paraformaldehyde, permeabilized with 0.1% Triton X-100, and blocked with 4% BSA. They were then incubated overnight at 4 °C with an αSMA antibody (R&D, USA), labeled with Alexa Fluor® secondary antibodies (LIANKE, China), and the slides were imaged using a microscope (ZEISS, Germany) with a 100× objective lens.

### 2.5 Cell growth assay

For this experiment, 2 × 10^3^ NFs/CAFs or HCC1937 cells in their corresponding medium were seeded into each well of 96-well plates. The adherent cells were maintained for 0, 2, 4 and 6 days, after which cell numbers were evaluated using Cell Counting Kit (CCK8) assays (APExBio, China). The medium devoid of cells served as a control. The absorbance of each well was measured at 450 nm, and growth curves were constructed based on the obtained data.

### 2.6 Chemotherapy sensitivity assay

Pretreated or untreated HCC1937 cells were seeded into 96-well plates at a density of 5 × 10^3^ cells per well. Following cell attachment, the cells were treated with varying concentrations of doxorubicin (0, 0.01, 0.02, 0.05, 0.1, 0.2, 0.5, 1 and 2 μg/ml). After a 24-h incubation period, the viability of the cells was assessed using CCK8 assays, the absorbance of each well was measured at 450 nm, and their corresponding survival rate curves were constructed based on the obtained data.

### 2.7 Migration and invasion assay

For in vitro invasion and migration assays, the indicated cells (20,000 cells/well for NFs or CAFs and 50,000 cells/well for HCC1937 cells) were seeded in a serum-free medium into uncoated transwell inserts (Corning, USA) or inserts coated with Matrigel (BD Biosciences, USA). The receiver plates were loaded with medium containing 20% FBS, and after incubation at 37 °C for 16 h, cells on the upper side of inserts were gently removed, while cells that had transversed the pores were fixed using 4% paraformaldehyde (Solarbio, China) for 20 min, then stained with 0.1% crystal violet (Solarbio, China) for 15 min. The inserts were washed with phosphate-buffered saline (PBS) and air-dried at room temperature, following which the inserts were assessed under an inverted microscope (ZEISS, Germany).

### 2.8 MicroRNA sequencing

Total RNA, including small RNAs, was isolated utilizing a commercial total RNA purification kit sourced from Norgen Biotek Corp (Thorold, ON, Canada). The quality and quantity of the extracted RNA were assessed using a Bioanalyzer 2100 (Agilent Technologies, USA). A small RNA library was generated from 1 μg of total RNA using a TruSeq small RNA sample preparation kit (Illumina, USA). After library preparation, single-end sequencing with a read length of 50 base pairs was executed on an Illumina HiSeq 2500 platform (Illumina, USA), following the manufacturer's instructions.

### 2.9 miRNA-seq data analysis

Raw reads were subjected to thorough analysis using our in-house program, ACGT101-miR (LC Sciences, Houston, Texas, USA), to eliminate adapter dimers, junk, low complexity reads, common RNA families (including rRNA, tRNA, snRNA and snoRNA), and repeats. Subsequently, unique sequences ranging from 18 to 26 nucleotides were compared with specific precursor species in miRBase 22.0 through a BLAST search to identify both known miRNAs and novel 3p- and 5p-derived miRNAs while allowing for a single mismatch in the sequence and accommodating length variations at the 3′ and 5′ ends in the alignment. For known miRNAs, unique sequences that aligned with specific mature miRNA species in hairpin arms were classified as known miRNAs. Novel 5p- or 3p-derived miRNA candidates were recognized as sequences mapping to the opposite arm of known specific hairpin precursor species containing annotated mature miRNA-containing arms. For further characterization, the remaining sequences were mapped to other selected precursor species (excluding specific species) in miRBase 22.0 through BLAST searches. The mapped pre-miRNAs were then subjected to additional BLAST analysis against specific genomes to pinpoint their genomic locations, which allowed us to define these two categories of miRNAs as known miRNAs. Lastly, the unmapped sequences underwent BLAST searches against specific genomes, and hairpin RNA structures containing these sequences were identified and predicted based on the flanking 80 nucleotide sequences using the RNAfold software (http://rna.tbi.univie.ac.at/cgi-bin/RNAfold.cgi).

### 2.10 Gene ontology (GO) enrichment and kyoto encyclopedia of genes and genomes (KEGG) pathway enrichment

To comprehensively understand the functions of the dysregulated miRNAs, we conducted GO analysis using the DAVID bioinformatics resource (version 6.8; https://david.ncifcrf.gov/). Additionally, for pathway enrichment analysis, we utilized the KEGG enrichment analysis through the KOBAS web server (version 3.0; http://kobas.cbi.pku.edu.cn/). Enrichment significance was determined at a threshold of *P* < 0.05.

### 2.11 Statistical analysis

All experiments were performed in triplicate and repeated thrice. The results are presented as mean ± standard deviation (SD). An unpaired two-tailed Student's t-test or one-way ANOVA test was applied to assess statistical significance to calculate *P* values, for which a significance level of *P* < 0.05 was considered statistically significant.

## 3 Results

### 3.1 Identification of NFs and CAFs from breast cancer tissues

Following isolation from normal breast and breast cancer tissues and subsequent culture in medium, a comparison of morphology and biomarkers between NFs and CAFs was conducted. Phase-contrast micrographs illustrated that CAFs exhibited a relatively elongated spindle shape (increased aspect ratio) compared to NFs (Fig. 2a), suggesting a heightened potential for migration and invasion. Both NFs and CAFs were found to form compact mammospheres under low-attachment conditions (Fig. 2b), which were then used for subsequent SEM and TEM analyses. The SEM images revealed the presence of villous structures across the surfaces of both NFs and CAFs and that CAFs exhibited a more pronounced elongated spindle shape in contrast to NFs (Fig. 2c). TEM analysis revealed numerous vesicular structures in both NFs and CAFs (Fig. 2d), indicative of secretory functions that could potentially influence neighboring cells. α-Smooth muscle actin (αSMA) is widely recognized as a prevalent biomarker in CAFs and has been commonly used in several studies [[13], [14], [15], [16]]. Thus, we also assessed the expression of αSMA in CAFs and NFs in the present study, using vimentin as a marker of mesenchymal origin. Western blot results consistently demonstrated overexpression of αSMA in CAFs when compared to NFs across all three pairs of primary fibroblast cells (Fig. 2e). Conversely, NFs and CAFs exhibited similar expression levels of vimentin, which is a typical fibroblast marker (Fig. 2e). Immunofluorescence analysis validated the higher expression of αSMA in CAFs (Fig. 2f). Consequently, CAFs and NFs isolated from breast cancer samples and normal breast tissues, respectively, exhibited minor distinctions in morphology alongside substantial differences in biomarker expression.

**Fig. 2:**
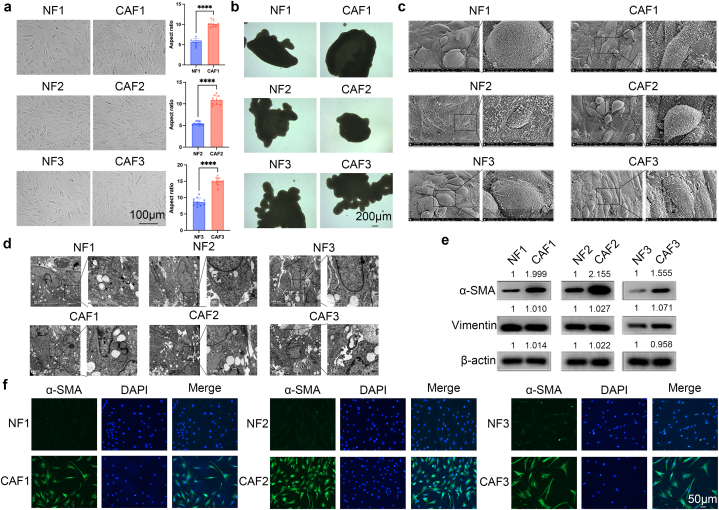
Differences between NFs and CAFs in terms of morphology and biomarkers. (a) Morphology of NFs and CAFs visualized under an optical microscope. Aspect ratio of CAFs and NFs are compared. (b) Morphology of mammospheres formed by NFs and CAFs under an optical microscope. (c) Morphology of NFs and CAFs under SEM. (d) Morphology of NFs and CAFs under TEM. (e) Expression of biomarkers in NFs and CAFs detected by Western blot. (f) Expression of α-SMA in NFs and CAFs detected by Immunofluorescence. Statistical analysis was performed using a two-tailed unpaired Student's t-test, where *p* < 0.05 indicated statistical significance. ****p < 0.0001.

### 3.2 The growth, migration and invasion abilities of CAFs were higher than those of NFs

**Fig. 3:**
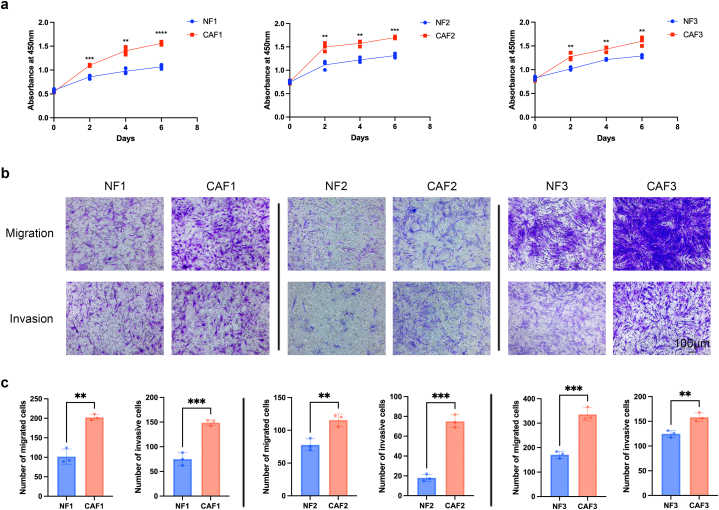
CAFs exhibited greater growth, migration and invasion capability than NFs. (a) Growth curve of NFs and CAFs assessed by CCK8. (b and c) Representative photomicrographs (b) and quantification (c) illustrating the migration and invasion capabilities of NFs and CAFs evaluated through transwell assay. Each experiment was replicated three times, and data are presented as mean ± SD. Statistical analysis was performed using a two-tailed unpaired Student's t-test, where *p* < 0.05 indicated statistical significance. **p < 0.01; ***p < 0.001; ****p < 0.0001.

To delineate the characteristics of CAFs and NFs, we evaluated their growth rate, migration and invasion. CCK8 assays revealed a significantly elevated growth rate across all three CAF cultures in comparison to the three NF cultures (Fig. 3a). Analysis of the transwell assay data revealed that all three CAF cultures exhibited greater migration and invasion capabilities in contrast to the three NF cultures (Fig. 3b and **c**). Thus, the CAFs and NFs isolated from the three sample pairs in our study showcased substantial variations in both biomarkers and malignant traits.

### 3.3 CAFs conferred higher growth, migration, invasion, and chemoresistance potential to breast cancer cells than those induced by NFs

**Fig. 4:**
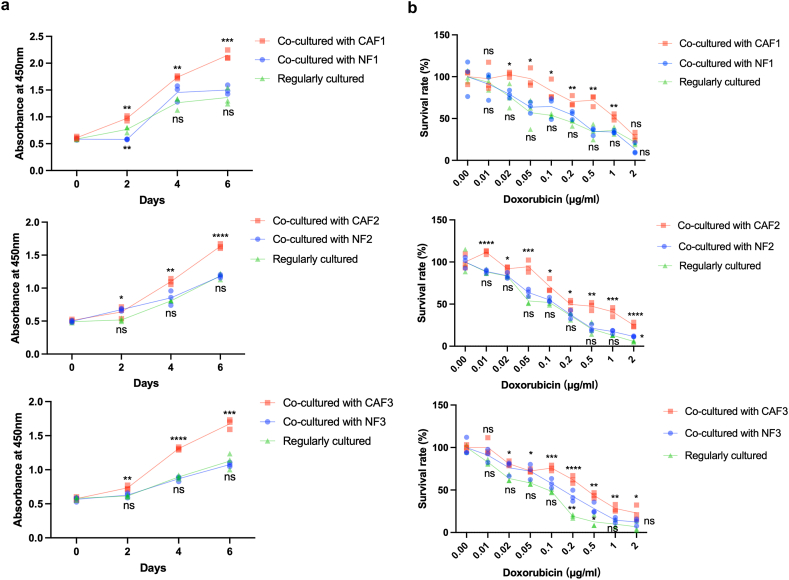
CAFs conferred more potential to breast cancer cells in growth and chemoresistance than NFs. (a) Impact of coculture with NFs or CAFs on the growth of HCC1937 breast cancer cells. (b) Influence of coculture with NFs or CAFs on the sensitivity of HCC1937 breast cancer cells to doxorubicin. Each experiment was replicated three times, and data are presented as mean ± SD. Statistical analysis was performed using a one-way ANOVA test, where *p* < 0.05 indicated statistical significance. *p < 0.05; **p < 0.01; ***p < 0.001; ****p < 0.0001.

**Fig. 5:**
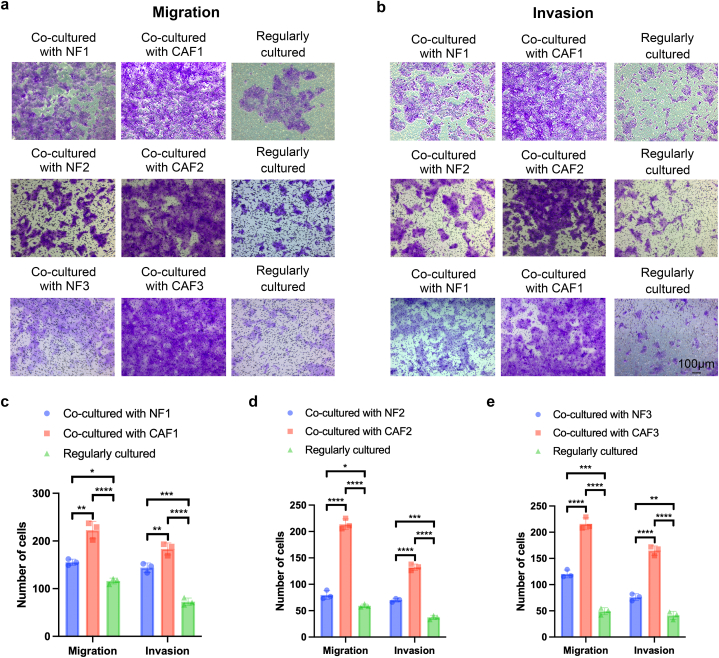
CAFs conferred more potential to breast cancer cells in migration and invasion than NFs. Representative photomicrographs (a, b) and quantification (c–e) depicting the migration (a) and invasion (b) under distinct coculture conditions for HCC1937 cells as assessed through transwell assay. Each experiment was replicated three times, and data are presented as mean ± SD. Statistical analysis was performed using a one-way ANOVA test, where *p* < 0.05 indicated statistical significance. *p < 0.05; **p < 0.01; ***p < 0.001; ****p < 0.0001.

To assess the effect of NFs and CAFs on breast cancer, we cocultured breast cancer HCC1937 cells with NFs or CAFs and compared the growth rate, migration, invasion and chemosensitivity with those in untreated breast cancer cells. The results showed that CAFs could significantly accelerate the growth of breast cancer cells compared to untreated cells, while no significant effects were observed with NFs (Fig. 4a). Similarly, CAFs considerably promoted the resistance to doxorubicin in breast cancer cells, while NFs demonstrated no effects (Fig. 4b). In contrast, NFs increased the migration and invasion capabilities of breast cancer cells, which were more pronounced in CAFs (Fig. 5a and b). Overall, CAFs demonstrated greater potential to enhance the malignant properties of breast cancer compared to NFs.

### 3.4 Identification and analysis of differentially expressed miRNAs in NFs and CAFs by RNA sequencing

**Fig. 6:**
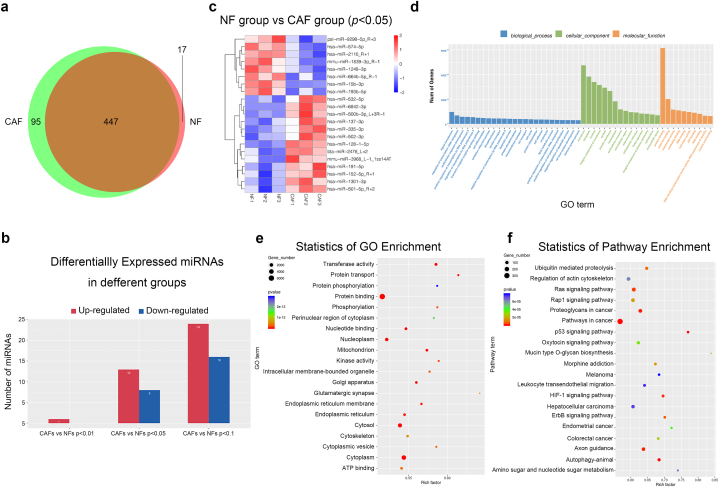
Identification and analysis of differentially expressed miRNAs in NFs and CAFs. (a) Venn diagram of detected miRNAs in the NF group and the CAF group. (b) Column diagram depicting the counts of differentially expressed miRNAs in the NF group and the CAF group at varying *p*-value thresholds of 0.01, 0.05, or 0.1. (c) Heat map of differentially expressed miRNAs in NFs and CAFs (*p* < 0.05). (d) Column diagram of GO analysis of target genes of differentially expressed miRNAs in NF group and CAF group. (e) Bubble chart of GO analysis of target genes of differentially expressed miRNAs in NF group and CAF group. f Bubble chart of KEGG analysis of target genes of differentially expressed miRNAs in NF group and CAF group.

Given the notable disparities observed between NFs and CAFs in terms of their influence on malignancy, we aimed to elucidate the underlying factors contributing to these differences. To this end, we conducted RNA sequencing to unveil the miRNA expression profile in both NFs and CAFs. The results identified 464 miRNAs in the 3 NF samples and 542 miRNAs in the 3 CAF samples. Among these, 447 miRNAs were found to be coexpressed across all 6 samples (Fig. 6a). A total of 21 miRNAs were identified as differentially expressed (13 upregulated and 8 downregulated) in CAFs versus NFs (*p* < 0.05) (Table 1). Adjustment of the p-value threshold to less than 0.01 and 0.1 resulted in the identification of 1 (upregulated) and 40 (24 upregulated and 16 downregulated) miRNAs with varying expression in CAFs versus NFs, respectively (Fig. 6b). A heatmap of differentially expressed miRNAs was then generated to visually represent these differential expressions (Fig. 6c). Next, GO analysis indicated a pronounced enrichment of target genes primarily associated with signal transduction in biological processes, membrane in cellular components, and protein binding in molecular functions (Fig. 6d). The top 20 GO enrichment genes are plotted in Fig. 6e. For an in-depth exploration of the functional implications, KEGG pathway enrichment analysis was performed, and the top 20 enriched pathways are depicted in Fig. 6f.

**Table 1:** Differentially expressed miRNAs in CAFs and NFs.

miRNA	miRNA sequence	CAFs/NFs (mean)	Fold change	p value (*t*-test)
hsa-miR-6842-3p	TTGGCTGGTCTCTGCTCCGCAG	Up	infinite	3.00E−02
hsa-miR-500b-3p_L+3R-1	AGTGCACCCAGGCAAGGATTCT	Up	7.330762	3.80E−02
mmu-miR-3968_L-1_1ss14AT	GAATCCCACTCCTGACACCA	Up	5.5261178	3.65E−02
hsa-miR-137-3p	TTATTGCTTAAGAATACGCGTAG	Up	4.22449926	4.05E−02
hsa-miR-335-3p	TTTTTCATTATTGCTCCTGACC	Up	3.37339905	4.15E−02
hsa-miR-152-5p_R+1	AGGTTCTGTGATACACTCCGACTC	Up	2.80557886	4.96E−02
bta-miR-2478_L+2	TCGTATCCCACTTCTGACACCA	Up	1.85633406	2.01E−02
hsa-miR-501-5p_R+2	AATCCTTTGTCCCTGGGTGAGAGT	Up	1.65312845	4.07E−02
hsa-miR-128-1-5p	CGGGGCCGTAGCACTGTCTGAGA	Up	1.62703782	7.17E−04
hsa-miR-532-5p	CATGCCTTGAGTGTAGGACCGT	Up	1.62566352	3.27E−02
hsa-miR-1301-3p	TTGCAGCTGCCTGGGAGTGACTTC	Up	1.58770036	1.83E−02
hsa-miR-502-3p	AATGCACCTGGGCAAGGATTCA	Up	1.45974347	4.29E−02
hsa-miR-191-5p	CAACGGAATCCCAAAAGCAGCTG	Up	1.42616655	2.85E−02
hsa-miR-2110_R+1	TTGGGGAAACGGCCGCTGAGTGA	Down	0.84403793	3.21E−02
hsa-miR-574-5p	TGAGTGTGTGTGTGTGAGTGTGT	Down	0.73242688	2.05E−02
hsa-miR-1249-3p	ACGCCCTTCCCCCCCTTCTTCA	Down	0.62039994	3.66E−02
hsa-miR-193b-5p	CGGGGTTTTGAGGGCGAGATGA	Down	0.59645372	2.41E−02
pal-miR-9298-5p_R+3	ACAGATGATGAACTTATTGACGGGCG	Down	0.52139857	3.79E−02
hsa-miR-664b-5p_R-1	TGGGCTAAGGGAGATGATTGGGT	Down	0.39261594	2.77E−02
hsa-miR-15b-3p	CGAATCATTATTTGCTGCTCTA	Down	0.30366196	1.42E−02
mmu-miR-1839-3p_R-1	AGACCTACTTATCTACCAACAG	Down	0.16017726	2.49E−02

**Fig. 7:**
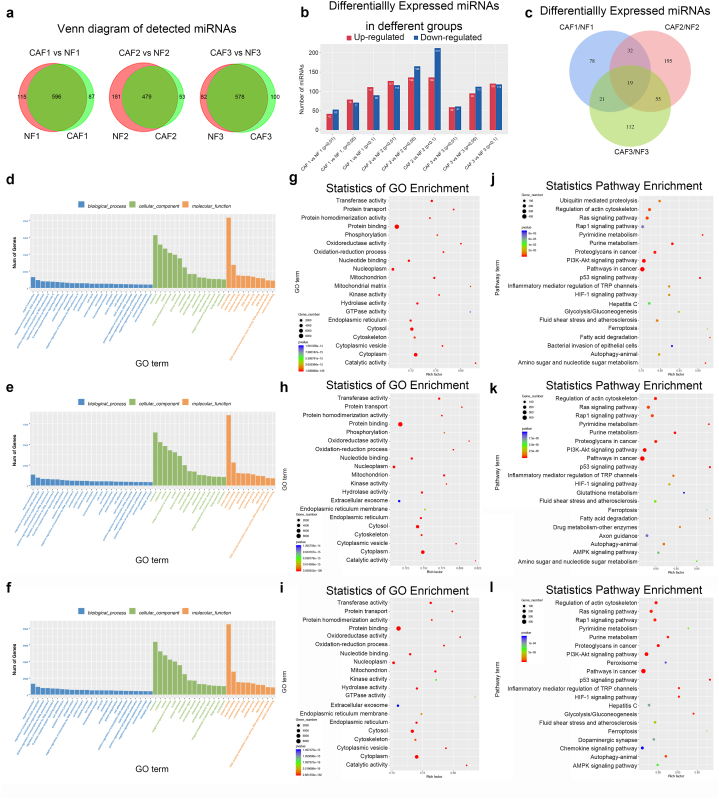
Comparison of differentially expressed miRNAs in each pair of NFs and CAFs. (a) Venn diagram of detected miRNAs in each pair of NFs and CAFs. (b) Column diagram of numbers of differentially expressed miRNAs in each pair of NFs and CAFs with *p*-value set as less than 0.01, 0.05 or 0.1. (c) Venn diagram of differentially expressed miRNAs in each pair of NFs and CAFs. (d–f) Column diagram of GO analysis of target genes of differentially expressed miRNAs in pair 1 (d), pair 2 (e) and pair 3 (f). g–i Bubble chart of GO analysis of target genes of differentially expressed miRNAs in pair 1 (g), pair 2 (h) and pair 3 (i). j–l Bubble chart of KEGG analysis of target genes of differentially expressed miRNAs in pair 1 (j), pair 2 (k) and pair 3 (l).

Due to the heterogeneity of the samples from different patients, each pair of CAFs and NFs were compared separately to acquire additional insights. A total of 596 of 798, 479 of 713, and 578 of 760 detected miRNAs were found to be coexpressed by NFs and CAFs in pairs 1, 2, and 3, respectively (Fig. 7a). The numbers of differentially expressed miRNAs in each pair are shown in Fig. 7b. When the p-value was set below 0.05, 150 (79 upregulated and 71 downregulated), 301 (136 upregulated and 165 downregulated), and 207 (95 upregulated and 112 downregulated) differentially expressed miRNAs were detected in pairs 1, 2, and 3. respectively (Fig. 7b), based on which subsequent analysis was performed. The results revealed 19 common differentially expressed miRNAs in 3 pairs of samples (Fig. 7c, Table S1). The corresponding GO and KEGG analyses of the target genes of differential miRNAs in each pair of samples are shown in Fig. 7d–l. Although the differential miRNAs were found to be largely different between the 3 pairs of samples (Table 2, Table 3, Table 4 and Fig. 7c), their target genes were enriched in similar GO terms and KEGG pathways (Table S2). Specifically, among the top 20 enriched GO terms and KEGG pathways, 17 and 14, respectively, were observed to overlap across all three pairs of samples. Heatmaps of the differential miRNAs in each pair of samples are shown in Fig. S1-3.

**Table 2:** Top 40 differentially expressed miRNA in CAF1 and NF1.

miRNA	miRNA Sequence (5′ to 3′)	CAF1/NF1	Fold change	p value (Chi square 2 × 2)	p value (Fisher test)
hsa-miR-346	TGTCTGCCCGCATGCCTGCCTCT	Down	0.09	5.23E−09	8.22E−10
hsa-miR-4485-5p_L+6R+1	AGAGGCACCGCCTGCCCAGTGAC	Down	0.10	5.64E−08	1.70E−08
hsa-mir-6511a-2-p5	CCTGCAGGCAGAAGTGGGGCTGA	Down	0.15	1.12E−03	7.29E−04
hsa-mir-6511b-1-p5	CCTGCAGGCAGAAGTGGGGCTGA	Down	0.15	1.12E−03	7.29E−04
ssc-mir-1285-p5	ATCGCGCCTGTGAATAGCCACTG	Down	0.18	9.56E−58	2.63E−63
bta-mir-2887-1-p3_1ss14AT	GTCCGGTGCGGAGTGCCCTTCGTCC	Down	0.21	1.12E−05	1.54E−05
hsa-miR-664b-5p_R-1	TGGGCTAAGGGAGATGATTGGGT	Down	0.23	1.89E−04	1.17E−04
hsa-mir-7110-p3_1ss18AC	CTCTCTCTCTCTCTCCCCC	Down	0.23	8.98E−08	6.22E−08
hsa-miR-16-2-3p_L+1R-1	ACCAATATTACTGTGCTGCTTT	Down	0.25	3.78E−04	3.13E−04
hsa-miR-15b-3p	CGAATCATTATTTGCTGCTCTA	Down	0.26	8.95E−03	1.18E−02
hsa-let-7a-3p_R+1	CTATACAATCTACTGTCTTTCC	Down	0.27	8.22E−10	8.22E−10
hsa-miR-191-3p	GCTGCGCTTGGATTTCGTCCCC	Down	0.28	1.70E−08	1.70E−08
hsa-miR-4728-3p	CATGCTGACCTCCCTCCTGCCCCAG	Down	0.31	7.29E−04	7.29E−04
hsa-miR-4524a-3p	TGAGACAGGCTTATGCTGCTAT	Down	0.31	7.29E−04	7.29E−04
hsa-miR-585-3p_R+3	TGGGCGTATCTGTATGCTAGGG	Down	0.32	2.63E−63	2.63E−63
hsa-mir-3138-p5	ACTTCCCCCACCTCACTGCCC	Down	0.32	1.54E−05	1.54E−05
hsa-miR-195-3p_R+1	CCAATATTGGCTGTGCTGCTCCA	Down	0.33	1.17E−04	1.17E−04
hsa-miR-5001-3p_R+1	TTCTGCCTCTGTCCAGGTCCTTT	Down	0.34	6.22E−08	6.22E−08
hsa-miR-146b-3p	GCCCTGTGGACTCAGTTCTGGT	Down	0.36	3.13E−04	3.13E−04
bta-miR-2887_R+4	CGGGACCGGGGTCCGGTGCGGAGT	Down	0.36	1.18E−02	1.18E−02
hsa-miR-500b-3p_L+3R-1	AGTGCACCCAGGCAAGGATTCT	Up	10.71	9.80E−09	8.18E−10
hsa-miR-935_L-1	CAGTTACCGCTTCCGCTACCGC	Up	9.41	1.79E−80	1.59E−92
hsa-miR-2682-5p	CAGGCAGTGACTGTTCAGACGTC	Up	7.38	4.81E−05	1.52E−05
hsa-miR-1246_L-1R+1	ATGGATTTTTGGAGCAGGG	Up	6.66	1.61E−04	4.91E−05
hsa-miR-137-5p	ACGGGTATTCTTGGGTGGATAAT	Up	6.16	1.29E−07	2.28E−08
pal-miR-9226-5p_L-3	AGTCCCTGTTCGGGCGCCA	Up	4.89	5.01E−03	4.42E−03
hsa-miR-335-3p	TTTTTCATTATTGCTCCTGACC	Up	4.81	1.68E−10	2.06E−11
hsa-miR-1908-5p	CGGCGGGGACGGCGATTGGTC	Up	4.28	8.40E−03	4.42E−03
eca-mir-8986b-p5_1ss1CG	GCCGAGACTAGAGTCACATCCTGAC	Up	4.06	9.91E−05	6.86E−05
hsa-miR-335-5p	TCAAGAGCAATAACGAAAAATGT	Up	4.01	8.12E−12	1.87E−12
hsa-miR-129-5p	CTTTTTGCGGTCTGGGCTTGC	Up	3.47	6.44E−04	6.78E−04
hsa-miR-487b-5p_L+1R-1	AGTGGTTATCCCTGTCCTGTTC	Up	3.33	3.87E−02	3.51E−02
hsa-miR-299-3p	TATGTGGGATGGTAAACCGCTT	Up	3.17	3.16E−02	3.09E−02
hsa-miR-137-3p	TTATTGCTTAAGAATACGCGTAG	Up	2.97	2.23E−03	3.37E−03
hsa-miR-181a-3p	ACCATCGACCGTTGATTGTACC	Up	2.87	2.12E−11	9.43E−12
PC-3p-40394_24	CGCCGCGCGCTTCCCTCCGCAT	Up	2.83	2.94E−02	2.66E−02
bta-miR-11980_R-1_1ss4CG	AGGGAACGGGCTTGGCGGA	Up	2.80	3.56E−28	2.87E−29
hsa-miR-495-3p_R+1	AAACAAACATGGTGCACTTCTTT	Up	2.73	3.00E−04	1.76E−04
hsa-miR-1228-3p_R+2	TCACACCTGCCTCGCCCCCCAA	Up	2.65	1.00E−02	1.35E−02
ssc-miR-30a-3p_R+1	CTTTCAGTCGGATGTTTGCAGCC	Up	2.49	2.92E−02	1.91E−02

**Table 3:** Top 40 differentially expressed miRNA in CAF2 and NF2.

miRNA	miRNA sequence (5′ to 3′)	CAF2/NF2	Fold change	p value (Chi square 2 × 2)	p value (Fisher test)
hsa-miR-126-3p	TCGTACCGTGAGTAATAATGCG	down	0.02	0.00E+00	2.00E−300
hsa-miR-451a_R-1	AAACCGTTACCATTACTGAGT	down	0.07	0.00E+00	0.00E+00
hsa-miR-889-3p	TTAATATCGGACAACCATTGT	down	0.13	1.35E−08	2.35E−08
mmu-mir-6240-p5_1ss23GA	GATTTCTGCCCAGTGCTCTGAAA	down	0.14	5.93E−09	3.40E−09
hsa-miR-106b-5p_R+1	TAAAGTGCTGACAGTGCAGATA	down	0.16	2.65E−25	8.16E−27
PC-3p-6433_288	TCGTGAAGCGTTCCATATTTTTT	down	0.17	5.49E−56	5.67E−59
mmu-miR-146a-5p_R+1	TGAGAACTGAATTCCATGGGTTT	down	0.17	1.12E−62	4.22E−66
hsa-miR-412-3p_L+2R-3	GTACTTCACCTGGTCCACTAGC	down	0.18	3.01E−08	8.52E−09
mmu-miR-2137_L-2R-1_1ss16AG	CGGCGGGAGCCCCGGGGA	down	0.19	3.58E−26	1.75E−27
hsa-miR-29c-3p	TAGCACCATTTGAAATCGGTTA	down	0.20	4.47E−07	4.34E−07
hsa-miR-146b-5p_R+1	TGAGAACTGAATTCCATAGGCTGT	down	0.20	8.02E−48	7.96E−50
hsa-miR-34c-3p	AATCACTAACCACACGGCCAGG	down	0.21	1.51E−54	1.08E−56
hsa-miR-378c_R-5	ACTGGACTTGGAGTCAGAAG	down	0.21	2.96E−08	1.38E−08
hsa-miR-491-5p_R+1	AGTGGGGAACCCTTCCATGAGGA	down	0.21	0.00E+00	3.91E−252
hsa-miR-17-3p	ACTGCAGTGAAGGCACTTGTAG	down	0.21	1.70E−06	1.45E−06
mmu-miR-218-1-3p	AAACATGGTTCCGTCAAGCACC	down	0.22	2.36E−67	6.92E−70
hsa-miR-146a-5p	TGAGAACTGAATTCCATGGGTT	down	0.23	0.00E+00	4.85E−238
hsa-miR-378a-5p	CTCCTGACTCCAGGTCCTGTGT	down	0.24	7.21E−26	2.54E−26
hsa-miR-3661_R+1	TGACCTGGGACTCGGACAGCTGT	down	0.24	2.01E−04	5.92E−04
bta-mir-2887-1-p3_1ss14AT	GTCCGGTGCGGAGTGCCCTTCGTCC	down	0.24	1.03E−13	2.84E−14
ssc-mir-1285-p5	ATCGCGCCTGTGAATAGCCACTG	up	293.92	0.00E+00	0.00E+00
PC-5p-1557_1535	AACCCGGTCAGCCCCTCTCCG	up	66.41	0.00E+00	2.06E−250
hsa-miR-543	AAACATTCGCGGTGCACTTCTT	up	17.91	2.92E−64	1.03E−80
mmu-mir-6240-p5_1ss15TG	CGGCGGGTGTTGACGCGATGTGATT	up	8.18	0.00E+00	0.00E+00
hsa-miR-224-5p_L-1	CAAGTCACTAGTGGTTCCGTTTAG	up	6.54	0.00E+00	0.00E+00
hsa-miR-500b-3p_L+3R-1	AGTGCACCCAGGCAAGGATTCT	up	5.91	4.19E−08	1.01E−08
hsa-miR-199a-5p	CCCAGTGTTCAGACTACCTGTTC	up	5.72	0.00E+00	0.00E+00
hsa-miR-199b-5p	CCCAGTGTTTAGACTATCTGTTC	up	4.85	2.34E−80	3.62E−89
hsa-miR-204-5p_R+1	TTCCCTTTGTCATCCTATGCCTG	up	4.82	3.31E−12	3.28E−13
PC-5p-3659_555	ATTCCCACTGTCCCTACCTATT	up	4.33	4.31E−15	2.42E−16
hsa-miR-122-5p_R-1	TGGAGTGTGACAATGGTGTTT	up	4.33	2.30E−06	7.65E−07
hsa-miR-3909	TGTCCTCTAGGGCCTGCAGTCT	up	4.23	6.01E−08	2.89E−08
hsa-miR-210-5p_R+1	AGCCCCTGCCCACCGCACACTGC	up	4.20	7.19E−05	5.87E−05
hsa-miR-335-3p	TTTTTCATTATTGCTCCTGACC	up	4.16	5.31E−10	9.54E−11
PC-5p-9926_172	TTAGTGGCTCCCTCTGCCTGCA	up	3.92	2.33E−04	1.97E−04
hsa-miR-145-3p	GGATTCCTGGAAATACTGTTCT	up	3.54	4.03E−04	3.34E−04
hsa-miR-365b-5p_R+1	AGGGACTTTCAGGGGCAGCTGTG	up	3.34	6.38E−17	5.99E−18
hsa-miR-542-5p	TCGGGGATCATCATGTCACGAGA	up	3.33	5.51E−03	7.37E−03
hsa-miR-4524a-3p	TGAGACAGGCTTATGCTGCTAT	up	2.99	1.63E−02	2.06E−02
hsa-miR-1292-5p_R-1	TGGGAACGGGTTCCGGCAGACGCT	up	2.95	8.72E−04	9.81E−04

**Table 4:** Top 40 differentially expressed miRNA in CAF3 and NF3.

miRNA	miRNA sequence (5′ to 3′)	CAF3/NF3	Fold change	p value (Chi square 2 × 2)	p value (Fisher test)
hsa-miR-142-5p_L+2R-3	CCCATAAAGTAGAAAGCACT	Down	0.07	9.57E−22	2.45E−25
hsa-miR-451a_R-1	AAACCGTTACCATTACTGAGT	Down	0.10	0.00E+00	0.00E+00
hsa-miR-122-5p_R-1	TGGAGTGTGACAATGGTGTTT	Down	0.11	2.13E−52	2.61E−60
hsa-miR-346	TGTCTGCCCGCATGCCTGCCTCT	Down	0.15	9.94E−09	4.63E−09
hsa-miR-1228-3p_R+2	TCACACCTGCCTCGCCCCCCAA	Down	0.18	3.25E−04	1.90E−04
hsa-miR-16-2-3p_L+1R-1	ACCAATATTACTGTGCTGCTTT	Down	0.19	7.44E−09	3.80E−09
hsa-miR-337-5p	GAACGGCTTCATACAGGAGTT	Down	0.22	7.26E−04	5.73E−04
hsa-miR-210-5p_R+1	AGCCCCTGCCCACCGCACACTGC	Down	0.22	1.22E−28	8.46E−31
hsa-miR-196a-5p	TAGGTAGTTTCATGTTGTTGGG	Down	0.23	3.75E−11	1.23E−11
hsa-miR-664b-5p_R-1	TGGGCTAAGGGAGATGATTGGGT	Down	0.24	4.26E−04	5.39E−04
hsa-miR-483-5p	AAGACGGGAGGAAAGAAGGGAG	Down	0.25	7.35E−03	7.36E−03
hsa-miR-937-3p	ATCCGCGCTCTGACTCTCTGCC	Down	0.25	7.35E−03	7.36E−03
PC-3p-34029_33	TTGCCTGCCCTCTTCCTCCAGT	Down	0.25	3.32E−03	4.20E−03
bta-miR-2478_L-2	ATCCCACTTCTGACACCA	Down	0.29	3.68E−76	1.89E−80
bta-mir-1246-p5_1ss17AG	GGATTTTTGGAGCAGGGAG	Down	0.30	1.45E−02	1.72E−02
hsa-mir-5100-p3_1ss17TC	ATCCCAGCGGTGCCTCCA	Down	0.31	1.59E−06	7.97E−07
pal-miR-9993b-3p	ATCTCGCTGGGGCCTCCA	Down	0.31	5.51E−20	5.80E−21
hsa-miR-129-5p	CTTTTTGCGGTCTGGGCTTGC	Down	0.31	5.26E−61	7.53E−64
hsa-miR-129-2-3p	AAGCCCTTACCCCAAAAAGCAT	Down	0.33	0.00E+00	2.58E−254
hsa-miR-377-3p	ATCACACAAAGGCAACTTTTGT	Down	0.33	1.68E−02	2.31E−02
mdo-miR-125b-5p_L+2_1ss7TG	AATCCCGGAGACCCTAACTTGTGA	Up	22.65	0.00E+00	1.42E−113
hsa-miR-504-5p	AGACCCTGGTCTGCACTCTATC	Up	11.96	9.31E−21	1.18E−23
hsa-miR-500b-3p_L+3R-1	AGTGCACCCAGGCAAGGATTCT	Up	8.14	9.59E−09	3.90E−09
hsa-miR-199b-5p	CCCAGTGTTTAGACTATCTGTTC	Up	7.58	1.08E−70	5.20E−79
bta-mir-2887-1-p5	GGACCGGGGTCCGGTGCGGAG	Up	5.44	4.62E−07	3.01E−07
hsa-miR-675-3p_R+2	CTGTATGCCCTCACCGCTCAGC	Up	5.30	8.53E−24	1.96E−25
hsa-miR-2682-3p_L+1R-1_1ss22CT	ACGCCTCTTCAGCGCTGTCTTT	Up	4.07	8.60E−04	1.24E−03
PC-5p-21047_68	CCGCCGCGGCGGCCGTCGGGT	Up	3.99	1.05E−03	1.24E−03
hsa-miR-452-5p_R+2	AACTGTTTGCAGAGGAAACTGAGA	Up	3.94	1.80E−07	1.23E−07
hsa-miR-3934-5p_R+1	TCAGGTGTGGAAACTGAGGCAGG	Up	3.89	4.56E−04	7.04E−04
mmu-miR-5100_L+1_1ss22TC	TTCGAATCCCAGCGGTGCCTCC	Up	3.77	1.14E−03	1.24E−03
hsa-miR-660-3p_R+1	ACCTCCTGTGTGCATGGATTAC	Up	3.43	1.44E−04	1.32E−04
bta-miR-2887_R+4	CGGGACCGGGGTCCGGTGCGGAGT	Up	3.12	1.74E−05	1.84E−05
hsa-miR-224-5p_L-1	CAAGTCACTAGTGGTTCCGTTTAG	Up	2.94	0.00E+00	4.23E−95
hsa-miR-616-5p_R+1	ACTCAAAACCCTTCAGTGACTTC	Up	2.91	4.98E−03	6.30E−03
hsa-miR-130b-3p	CAGTGCAATGATGAAAGGGCAT	Up	2.89	9.82E−03	1.14E−02
hsa-miR-7976_R+3	TGCCCTGAGACTTTTGCTCTAA	Up	2.71	2.84E−02	4.92E−02
hsa-miR-421	ATCAACAGACATTAATTGGGCGC	Up	2.69	2.81E−04	3.48E−04
hsa-miR-218-5p_R+1	TTGTGCTTGATCTAACCATGTG	Up	2.64	1.22E−02	1.48E−02
hsa-miR-29c-5p_L-2R+3	ACCGATTTCTCCTGGTGTTCAGA	up	2.64	7.37E−03	8.21E−03

## 4 Discussion

Although CAFs are known to be fibroblast cells that proliferate within tumor tissues, this particular cellular subset remains inadequately characterized owing to its pronounced heterogeneity and the absence of distinct markers [17], and the differences in the morphology and biomarker between NFs and CAFs remain elusive [[18], [19], [20]]. Notably, α-smooth muscle actin (αSMA) has been extensively employed as a hallmark marker for identifying CAFs across various studies [[14], [15], [16]], and our current study's data corroborates this notion by revealing an evident overexpression of αSMA in CAFs. CAFs have been proven to deliver miRNA, DNA and proteins to surrounding tumor cells via exosomes [21]. Accordingly, plenty of vesicular structures were observed in NFs and CAFs with electron microscope (Fig. 2d), implying exosome secreting potential of NFs and CAFs.

CAFs have been proven to have increased proliferation [[22], [23], [24]], motility [22,23,25] and chemoresistance [26]. In the present study, we assessed the growth rate, migration and invasion of fibroblasts in breast cancer, and confirmed that CAFs have more pronounced malignancy-associated properties than NFs.

CAFs are believed to promote proliferation [27,28], metastasis [29], stemness [30] and treatment resistance [31] of cancer cells in various types of cancer. To assess the influence of NFs and CAFs on breast cancer cells, we established a coculture system using transwell inserts. The results revealed that coculture with CAFs significantly upregulated the growth rate and chemoresistance of breast cancer cells, whereas these effects were not evident in the presence of NFs. However, although NFs demonstrated a certain degree of migration and invasion on breast cancer cells, the extent was significantly lower than that detected with CAFs. The results of previous literature have been contradictory in regard to the role of NFs. Some studies demonstrated a suppressive role of NFs in cancer [[32], [33], [34]], while others showed that NFs played promoting roles [35].

This study successfully revealed substantial disparities in the phenotypes of NFs and CAFs. However, prior investigations have indicated that cancer-promoting phenotypes in CAFs are not primarily attributed to loss of heterozygosity (LOH) or copy number alterations, as these events are relatively infrequent [36,37]. On the contrary, variations in gene regulation at the transcriptional and posttranscriptional levels have been documented [9,10,12,38]. Thus, our study focused on the posttranscriptional regulation in this field. The result of RNA sequencing revealed significant differences in miRNA expression between NFs and CAFs. It has also been suggested that protumorigenic functions of CAFs are generally driven by their altered secretome [39], and microRNAs are important regulators of signaling in the TME [40], consistent with the data of our study obtained in an indirect coculture system. It should be noted that our research primarily focuses on in vitro observations and simplified experimental approaches, which may not fully encapsulate the intricate biology of CAFs within breast cancer tissue.

A total of 21 differentially expressed miRNAs were identified in the CAF group compared to the NF group and did not overlap with the dysregulated miRNAs previously reported in CAFs of breast cancer [38], implying considerable heterogeneity between NFs and CAFs in patients with breast cancer. Given this heterogeneity, we performed individual comparisons for each pair of CAFs and NFs. Intriguingly, among the three pairs of samples, we found that only 19 dysregulated miRNAs were shared, which could be attributed to variations in breast cancer subtypes and the inherent heterogeneity across patients. Nevertheless, despite the diversity observed, a remarkable similarity was found in the enrichment of target genes influenced by these dysregulated miRNAs, as indicated by GO terms and KEGG pathways, thereby implying that distinct posttranscriptional regulatory mechanisms might lead to similar phenotypic traits in CAFs. An integrative examination encompassing genomic and epigenomic regulation of the transcriptome, along with proteomic analyses, could help provide more detailed characterization of the gene expression profiles in CAFs and NFs, as well as valuable insights into deciphering the mechanisms underlying the cancer-promoting effects of CAFs.

## Ethics committee approval and patient consent

The research was approved by the Ethics Committee of Affiliated Cixi Hospital, Wenzhou Medical University. The ethics approval number is 2020-LP-KY002. Written informed consent was obtained from the patients.

## Author contribution statement

Dengdi Hu; Wenying Zhuo; Feiyang Ji; Xun Zhang; Yongxia Chen: Performed the experiments; Analzyed and interpreted the data.

Peirong Gong; Misha Mao; Yuehong Pan; Siwei Ju: Performed the experiments; Analyzed and interpreted the data; Wrote the paper.

Jun Shen: Conceived and designed the experiments; Contributed reagents, materials, analysis tools or data.

## Funding

The work was supported by 10.13039/100007834Ningbo Natural Science Foundation (Grant No. 2019A610315) and Cixi Agricultural and Social Development Science and Technology Project (CN2020006).

## Data availability statement

Data included in article/supp. material/referenced in article.

## Declaration of competing interest

The authors declare that they have no known competing financial interests or personal relationships that could have appeared to influence the work reported in this paper.

## Figures and Tables

**figs1:**
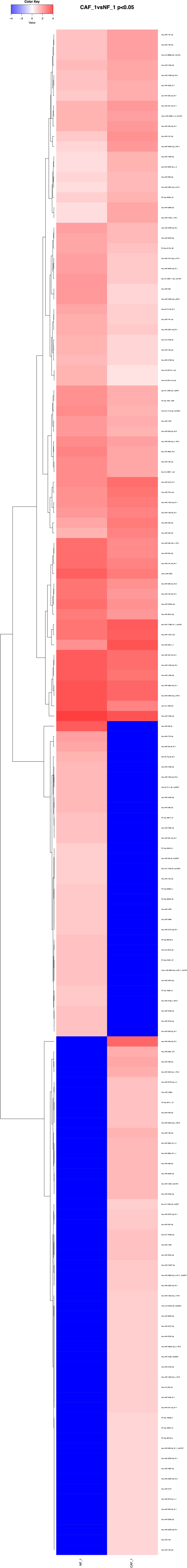


**figs2:**
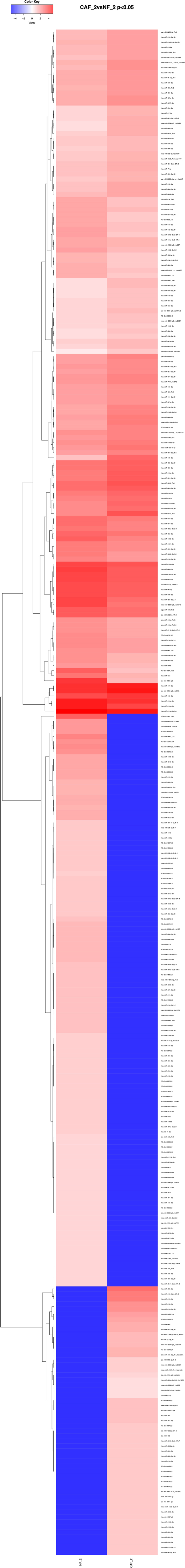


**figs3:**