# Supplementary material for: Biological differences between normal and cancer-associated fibroblasts in breast cancer
Source: Heliyon. 2023 Sep 6;9(9):e19803. doi: 10.1016/j.heliyon.2023.e19803 (PMC10559169; doi:10.1016/j.heliyon.2023.e19803)
Supplement: Multimedia component 1 [file mmc1.docx]

**Table S1. Overlapped differentially regulated miRNAs in 3 pairs of CAFs and NFs**

| miRNA | miRNA sequence | Fold change  (CAF1/NF1) | Fold change  (CAF2/NF2) | Fold change  (CAF3/NF3) |
| --- | --- | --- | --- | --- |
| hsa-miR-451a_R-1 | AAACCGTTACCATTACTGAGT | 2.23 | 0.072 | 0.1 |
| hsa-miR-346 | TGTCTGCCCGCATGCCTGCCTCT | 0.09 | -infinite | 0.15 |
| hsa-miR-1228-3p_R+2 | TCACACCTGCCTCGCCCCCCAA | 2.65 | infinite | 0.18 |
| hsa-miR-483-5p | AAGACGGGAGGAAAGAAGGGAG | infinite | infinite | 0.25 |
| hsa-miR-129-2-3p | AAGCCCTTACCCCAAAAAGCAT | 2.25 | 0.25 | 0.33 |
| hsa-miR-642a-5p | GTCCCTCTCCAAATGTGTCTTG | infinite | infinite | 0.43 |
| hsa-miR-95-3p_R-1 | TTCAACGGGTATTTATTGAGC | -infinite | infinite | infinite |
| hsa-miR-136-3p | CATCATCGTCTCAAATGAGTCT | infinite | infinite | infinite |
| hsa-miR-191-3p | GCTGCGCTTGGATTTCGTCCCC | 0.28 | infinite | -infinite |
| hsa-miR-192-5p | CTGACCTATGAATTGACAGCC | infinite | infinite | -infinite |
| hsa-miR-363-3p_R-1 | AATTGCACGGTATCCATCTGT | infinite | -infinite | -infinite |
| hsa-miR-3661_R+1 | TGACCTGGGACTCGGACAGCTGT | infinite | 0.24 | -infinite |
| hsa-miR-6842-3p | TTGGCTGGTCTCTGCTCCGCAG | infinite | infinite | infinite |
| hsa-mir-7110-p3_1ss18AC | CTCTCTCTCTCTCTCCCCC | 0.23 | infinite | -infinite |
| PC-5p-42917_21 | TCAGACACAGGTATGGCTGGCTCC | -infinite | -infinite | infinite |
| hsa-miR-137-3p | TTATTGCTTAAGAATACGCGTAG | 2.97 | infinite | 2.15 |
| ssc-mir-1285-p5 | ATCGCGCCTGTGAATAGCCACTG | 0.18 | 293.92 | 2.48 |
| hsa-miR-335-3p | TTTTTCATTATTGCTCCTGACC | 4.81 | 4.17 | 2.56 |
| hsa-miR-500b-3p_L+3R-1 | AGTGCACCCAGGCAAGGATTCT | 10.71 | 5.88 | 8.14 |

**Table S2. Common enrichment in GO terms and KEGG pathways in different pairs of samples**

| Enrichment | CAF1/NF1 | CAF2/NF2 | CAF3/NF3 | CAF group/NF group |
| --- | --- | --- | --- | --- |
| GO terms | protein binding | protein binding | protein binding | protein binding |
|  | cytoplasm | cytoplasm | cytoplasm | cytoplasm |
|  | cytosol | cytosol | cytosol | cytosol |
|  | transferase activity | transferase activity | transferase activity | transferase activity |
|  | mitochondrion | mitochondrion | mitochondrion | mitochondrion |
|  | catalytic activity | hydrolase activity | hydrolase activity | protein transport |
|  | hydrolase activity | oxidoreductase activity | catalytic activity | nucleotide binding |
|  | oxidoreductase activity | catalytic activity | oxidoreductase activity | nucleoplasm |
|  | protein transport | protein transport | protein transport | Golgi apparatus |
|  | nucleotide binding | endoplasmic reticulum | endoplasmic reticulum | endoplasmic reticulum |
|  | oxidation-reduction process | oxidation-reduction process | nucleotide binding | endoplasmic reticulum membrane |
|  | nucleoplasm | cytoplasmic vesicle | oxidation-reduction process | kinase activity |
|  | cytoplasmic vesicle | nucleotide binding | cytoplasmic vesicle | intracellular membrane-bounded organelle |
|  | endoplasmic reticulum | nucleoplasm | nucleoplasm | phosphorylation |
|  | kinase activity | protein homodimerization activity | protein homodimerization activity | cytoplasmic vesicle |
|  | cytoskeleton | cytoskeleton | cytoskeleton | ATP binding |
|  | mitochondrial matrix | kinase activity | GTPase activity | glutamatergic synapse |
|  | protein homodimerization activity | phosphorylation | endoplasmic reticulum membrane | cytoskeleton |
|  | phosphorylation | endoplasmic reticulum membrane | kinase activity | perinuclear region of cytoplasm |
|  | GTPase activity | extracellular exosome | extracellular exosome | protein phosphorylation |
| KEGG pathways | Pathways in cancer | Pathways in cancer | Pathways in cancer | p53 signaling pathway |
|  | PI3K-Akt signaling pathway | p53 signaling pathway | p53 signaling pathway | Autophagy - animal |
|  | p53 signaling pathway | PI3K-Akt signaling pathway | PI3K-Akt signaling pathway | Pathways in cancer |
|  | Purine metabolism | Purine metabolism | Proteoglycans in cancer | Proteoglycans in cancer |
|  | Proteoglycans in cancer | Regulation of actin cytoskeleton | Regulation of actin cytoskeleton | Axon guidance |
|  | Pyrimidine metabolism | Pyrimidine metabolism | Ras signaling pathway | HIF-1 signaling pathway |
|  | Amino sugar and nucleotide sugar metabolism | Proteoglycans in cancer | Glycolysis / Gluconeogenesis | Ras signaling pathway |
|  | Ras signaling pathway | Ras signaling pathway | Rap1 signaling pathway | ErbB signaling pathway |
|  | Regulation of actin cytoskeleton | Rap1 signaling pathway | HIF-1 signaling pathway | Ubiquitin mediated proteolysis |
|  | Fatty acid degradation | Inflammatory mediator regulation of TRP channels | Inflammatory mediator regulation of TRP channels | Morphine addiction |
|  | Ubiquitin mediated proteolysis | Autophagy - animal | Purine metabolism | Rap1 signaling pathway |
|  | Fluid shear stress and atherosclerosis | Drug metabolism - other enzymes | Autophagy - animal | Mucin type O-glycan biosynthesis |
|  | HIF-1 signaling pathway | Fatty acid degradation | Ferroptosis | Colorectal cancer |
|  | Inflammatory mediator regulation of TRP channels | HIF-1 signaling pathway | Fluid shear stress and atherosclerosis | Oxytocin signaling pathway |
|  | Ferroptosis | Fluid shear stress and atherosclerosis | Pyrimidine metabolism | Endometrial cancer |
|  | Autophagy - animal | Amino sugar and nucleotide sugar metabolism | AMPK signaling pathway | Regulation of actin cytoskeleton |
|  | Glycolysis / Gluconeogenesis | Ferroptosis | Hepatitis C | Amino sugar and nucleotide sugar metabolism |
|  | Hepatitis C | AMPK signaling pathway | Dopaminergic synapse | Hepatocellular carcinoma |
|  | Rap1 signaling pathway | Axon guidance | Peroxisome | Leukocyte transendothelial migration |
|  | Bacterial invasion of epithelial cells | Glutathione metabolism | Chemokine signaling pathway | Melanoma |
