# Supplementary figures and images for: Biological differences between normal and cancer-associated fibroblasts in breast cancer
Source: Heliyon. 2023 Sep 6;9(9):e19803. doi: 10.1016/j.heliyon.2023.e19803 (PMC10559169; doi:10.1016/j.heliyon.2023.e19803)

## Slide 1
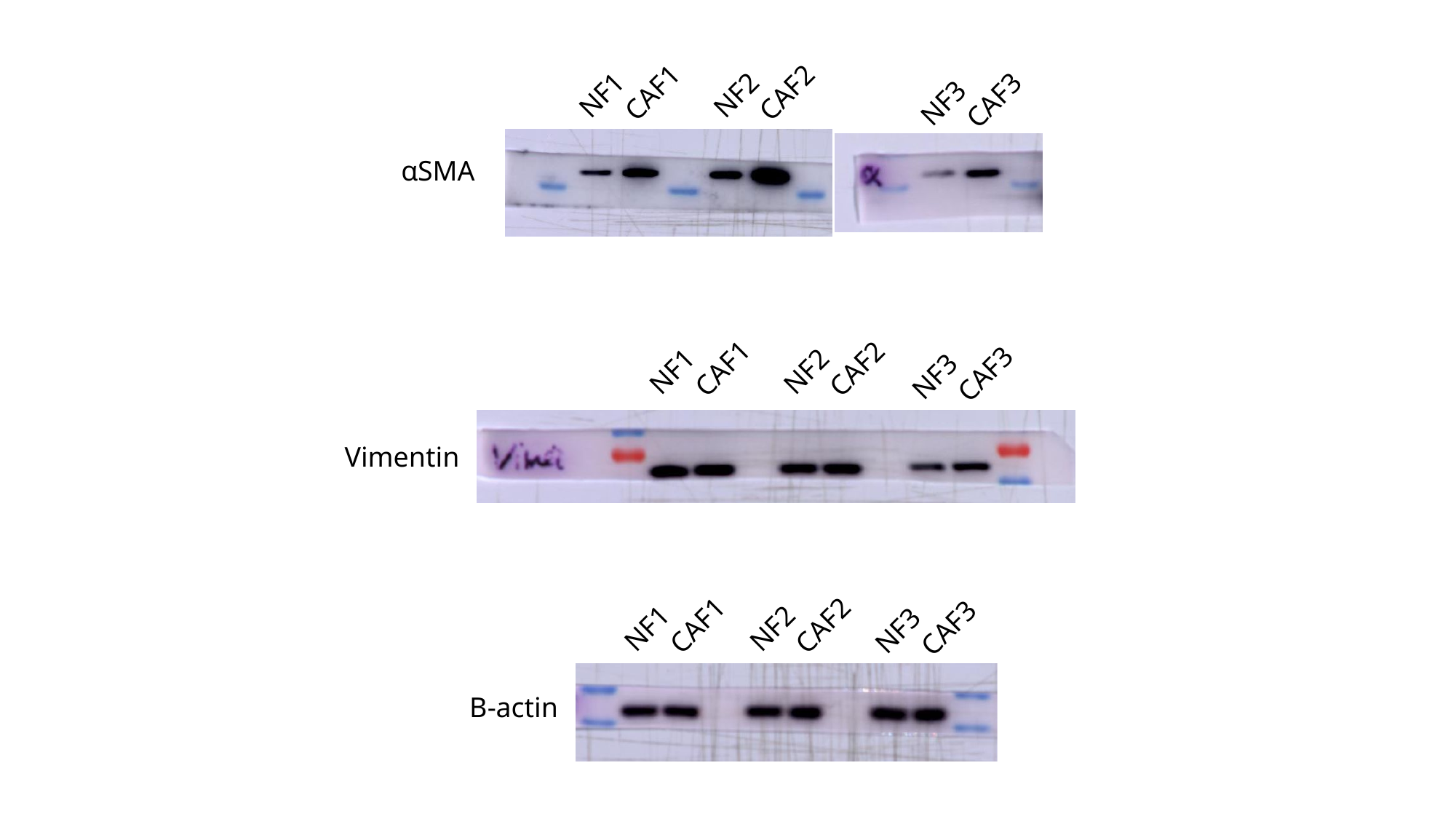

CAF1
CAF2
NF1
NF2
CAF3
NF3
αSMA
CAF1
CAF2
NF1
NF2
CAF3
NF3
Vimentin
CAF1
CAF2
CAF3
NF1
NF2
NF3
Β-actin

Supplement: Multimedia component 2 [file mmc2.pptx]
